# Peroxisome Proliferator-Activated Receptors Family Is Involved in the Response to Treatment and Mild Clinical Course in Patients with Ulcerative Colitis

**DOI:** 10.1155/2014/932530

**Published:** 2014-12-08

**Authors:** J. K. Yamamoto-Furusho, M. Jacintez-Cazares, J. Furuzawa-Carballeda, G. Fonseca-Camarillo

**Affiliations:** ^1^Inflammatory Bowel Disease Clinic, Department of Gastroenterology, Instituto Nacional de Ciencias Médicas y Nutrición, Salvador Zubirán, Vasco de Quiroga No. 15, Colonia Sección XVI, 14000 Mexico City, Mexico; ^2^Department of Immunology and Rheumatology, Instituto Nacional de Ciencias Médicas y Nutrición, Salvador Zubirán, Vasco de Quiroga No. 15, Colonia Sección XVI, 14000 Mexico City, Mexico

## Abstract

*Background*. PPARs play an important role in the regulation of intestinal inflammation. *Methods*. We included a total of 46 UC patients and 31 controls. The gene expression of PPARs was measured by RT-PCR and protein expression by immunohistochemistry. *Results*. PPAR*α* gene expression was significantly decreased in patients with active UC compared with remission UC group (*P* = 0.001) and controls (*P* = 0.001). We found that low gene expression of PPAR*α* in mucosa confers a higher risk of UC activity (*P* ≤ 0.0001, OR = 22.6). We observed an increase of PPAR*α* expression in patients with UC who were treated with 5-aminosalicylates compared with those who received any other combined therapy (*P* = 0.03, OR = 0.08). PPAR*γ* gene expression was decreased in the active UC group compared with UC in remission (*P* = 0.001) and control group (*P* = 0.001). An increased expression of PPAR*γ* gene was associated with mild clinical course of the disease (*P* ≤ 0.001, OR = 0.05). No gene expression of PPAR*β*/*δ* was found in the colonic mucosa from UC patients and controls. *Conclusion*. Our results suggest that patients with high gene expression of PPARs have a better response to medical treatment and a mild clinical course of the disease.

## 1. Introduction

The inflammatory bowel disease (IBD) comprises two types of chronic intestinal disorders: Crohn's disease (CD) and ulcerative colitis (UC). Accumulating evidence suggests that IBD results from an inappropriate inflammatory response to intestinal microbes in a genetically susceptible host [1].

The typical Western diet, which is high in fat and refined carbohydrates and low in fiber, has been linked to many chronic illnesses, including IBD [2]. Dietary and endogenously modified lipids modulate inflammation by functioning as intra- and intercellular signaling molecules. Proinflammatory lipid mediators such as the eicosanoids compete against the signaling actions of newly discovered modified fatty acids that act to resolve inflammation. In IBD multiple aberrancies in lipid metabolism have been discovered, which shed further light on the pathogenesis of intestinal inflammation [3].

PPARs function as regulators of lipid and lipoprotein metabolism and glucose homeostasis and influence cellular proliferation, differentiation, apoptosis, and inflammation. Peroxisome proliferator-activated receptors (PPARs) are ligand-activated transcription factors belonging to the nuclear receptor family; fatty acids and eicosanoids are natural PPAR ligands. The three peroxisome proliferator-activated receptors (PPARs), PPAR*α*, *β*, and *γ*, are derived from the nuclear hormone receptor gene family.

PPAR*α* is highly expressed in tissues such as liver, muscle, kidney, and heart, where it stimulates the beta-oxidative degradation of fatty acids. PPAR*γ* is predominantly expressed in intestine and adipose tissue. PPAR*γ* triggers adipocyte differentiation and promotes lipid storage. In addition, PPARs play an important role in inflammation control. PPAR activators inhibit the stimulation of proinflammatory response genes by negatively interfering with the NF-*κ*B and AP-1 signaling pathways. PPAR activators exert these anti-inflammatory activities in different immunological and vascular wall cell types such as monocyte-macrophages, endothelial, epithelial, and smooth muscle cells in which PPARs are expressed. These findings indicate a modulatory role for PPARs in the control of the inflammatory response with therapeutic applications in inflammation-related diseases such as UC [3, 4]. The gene expression of PPAR's family receptors has not been characterized in patients with UC. This paper is specifically focused on the role of PPARs gene and protein expression and its possible clinical contribution in UC patients.

## 2. Materials and Methods

### 2.1. Study Subjects

A total of 46 patients with diagnosis of UC were included during the period from January 2013 to January 2014 belonging to the Inflammatory Bowel Disease Clinic at the Instituto Nacional de Ciencias Médicas y Nutrición Hospital. The diagnosis of UC was done by the presence of the following criteria: a history of diarrhea or blood in stools, macroscopic appearance by endoscopy and biopsy compatible with UC. Relevant clinical and demographic information in all UC patients was collected from medical records: gender, age at diagnosis, familial aggregation, smoking history, previous appendectomy, disease evolution, extension, extraintestinal manifestations, medical or surgical treatment, and clinical course of disease. Colonoscopy was performed in order to calculate the Mayo Score Activity Index and take colonic biopsies. The clinical course of disease was defined as active then inactive (first episode with activity and then long-term remission for more than 5 years); intermittent activity (2 relapses in a year); chronic continual activity (>2 relapses or persistent activity despite medical conventional therapy) as previously described [5–7]. Exclusion criteria included patients with indeterminate colitis, Crohn disease, postradiation colitis, and infectious colitis. Control group consisted of noninflamed colonic biopsies (without endoscopic evidence of any type of colitis, neoplasia, or any other documented disease). Controls were matched by age and sex with patients.

### 2.2. Sample Processing and Gene Expression Analysis

All intestinal mucosal biopsies taken from colonoscopy were immediately placed in RNA later (Ambion, Austin, TX, USA) and stored at −70°C until processing.

Then total RNA was isolated using High Pure RNA Tissue (Roche Diagnostics, Mannheim, Germany), following the manufacturer's guidelines. Two hundred nanograms of total RNA was reverse-transcribed into cDNA with random hexamer primers (Roche Diagnostics, Mannheim, Germany).

The PPARs gene expression was measured by real-time polymerase chain reaction (RT-PCR). Expression of glyceraldehyde-3-phosphate dehydrogenase (GAPDH), a housekeeping gene, was analyzed for normalization purposes and quality controls. We used the following primers: PPAR*α* forward: 5′-gcactggaactggatgacag-3′ and reverse: 5′-tttagaaggccaggacgatct-3′, PPAR*β* forward: 5′-gggaaaagttttggcaggaand-3′, reverse: 5′-tgcccaaaacactgtacaaca-3′, PPAR*γ* forward: 5′-gacaggaaagacaacagacaaatc-3′, and reverse: 5′-ggggtgatgtgtttgaacttg-3′, GADPH forward: 5′-gcccaatacgaccaaatcc-3′, and reverse: 5′-agccacatcgctcagaca-3′, for normalization. PCR amplification of the abovementioned genes was carried out with 20 ng of cDNA, 200 nM forward and reverse primer, and Taqman Master Mix (Roche Diagnostics, Mannheim, Germany Roche Diagnostics, Mannheim, Germany) in a final volume of 10 *μ*L. PCR reactions were run in a Light Cycler 2.0 (Roche Diagnostics, Mannheim, Germany) for 45 cycles, each cycle consisting of denaturation for 15 seconds at 95°, primer annealing for 15 seconds at 55°, and extension for 30 seconds at 72°C and cooling 30 seconds at 40°C.

### 2.3. Immunohistochemistry

Paraffin-embedded-tissue slides were used for immunohistochemical staining with a mouse monoclonal anti-human PPAR*α* antibody, a mouse monoclonal anti-human PPAR*β* antibody, or a mouse monoclonal anti-human PPAR*γ* antibody (Santa Cruz Biotechnology, Santa Cruz, CA), respectively. The paraffin on a series of 5 mm sections was removed using xylene. The sections were then rehydrated using a graded ethanol series. Briefly, after deparaffinizing and demasking of antigens, the slides were then soaked in 90 mL of methanol/10 mL 30% H_2_O_2_ for 10–15 min at room temperature to block endogenous peroxidases and then washed with PBS. Slides were blocked with 10% normal serum and were incubated with avidin and biotin. Following incubation with the primary antibody (Santa Cruz Biotechnology, Santa Cruz, CA) overnight at 4°C, slides were incubated with the secondary, biotin-conjugated antibody. Slides were treated consecutively with HRP-conjugated goat anti-mouse IgG (Santa Cruz Biotechnology, Santa Cruz, CA) (diluted 1 : 2000) and incubated for 2 hr at room temperature. Next, the slides were incubated using a 3′-diaminobenzidine (DAB) substrate and the color reaction was allowed to develop for 5–10 min. After the staining reaction, the slides were washed thoroughly in tap water, counterstained with Mayer's hematoxylin, and mounted with cover slides. In the negative controls, cells were stained omitting the primary antibody.

### 2.4. Ethical Considerations

This work was performed according to the principles expressed in the Declaration of Helsinki. The study was approved by the ethical committee in our institution and a written informed consent was obtained from all patients.

### 2.5. Statistical Analysis

Statistical analysis was performed using the SigmaStat11.2 program (Aspire Software International, Leesburg, VA, USA) by the Kruskal-Wallis, One-Way Analysis of Variance on Ranks by Holm-Sidak method for all pairwise multiple comparison procedures. Data were expressed as the median, range, and mean ± SEM. A *P* value ≤ 0.05 was considered as significant.

## 3. Results

We studied a total of 46 patients with UC (22 men and 24 women) and 31 controls without evidence of inflammation (12 men and 19 women). Demographic data and disease characteristics from these patients are presented in Table 1.

### 3.1. Gene Expression of the PPAR Family in Patients with Ulcerative Colitis and Controls

**Figure 1:**
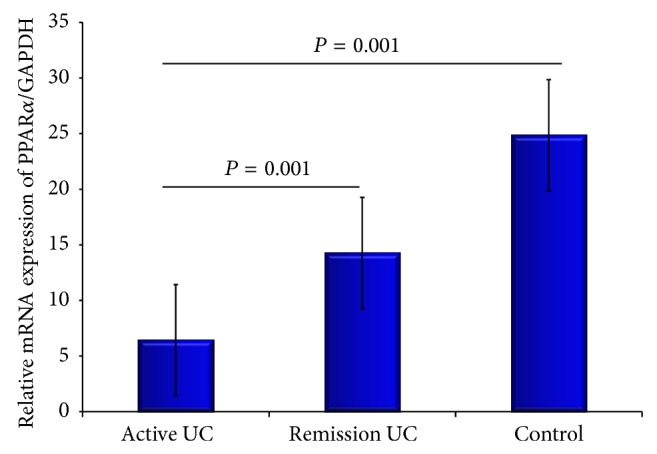
PPAR*α* expression was significantly decreased in patients with active UC compared with the remission group (*P* = 0.001) and controls (*P* = 0.001). Bars show means ± standard error of the mean of PPAR*α* transcript levels with GAPDH as housekeeping gene determined by 2^−ΔΔ^Ct. Differences among groups were assessed by Kruskal Wallis test.

PPAR*α* gene expression was significantly decreased in patients with active UC compared with remission group (*P* = 0.001) and controls (*P* = 0.001) (Figure 1). We found that low gene expression confers a higher risk of UC activity (*P* ≤ 0.001, OR = 22.6). We observed an increase of PPAR*α* gene expression in patients with UC who received only 5-aminosalicylates for treatment compared with those under combination therapy (*P* = 0.03, OR = 0.08).

**Figure 2:**
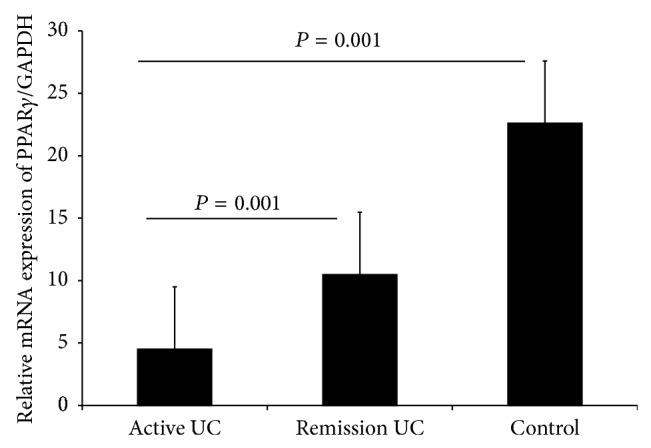
PPAR*γ* expression was significantly decreased in patients with active UC compared with the remission group (*P* = 0.001) and controls (*P* = 0.001). Bars show means ± standard error of the mean of PPAR*γ* transcript levels with GAPDH as housekeeping gene determined by 2^−ΔΔ^Ct. Differences among groups were assessed by Kruskal Wallis test.

We also found that PPAR*γ* gene expression was decreased in the active UC group compared to UC in remission (*P* = 0.01) and normal control groups (*P* ≤ 0.001) (Figure 2). Thus, an increased expression of this gene was associated with a protective and benign clinical course of the disease characterized for initial activity and then long-term remission more than 5 years (*P* ≤ 0.001, OR = 0.05).

We did not find gene expression of PPAR*β* in colonic mucosa from UC patients and controls.

### 3.2. Protein Expression of the PPAR Family in Patients with UC and Controls

In order to determine* in situ* PPAR*α*, PPAR*β*, and PPAR*γ* protein expression in intestinal biopsies from active UC patients, tissues were immunostained and compared with controls (noninflamed tissue).

PPARs immunoreactive cell percentage was higher in active UC compared with controls.

**Figure 3:**
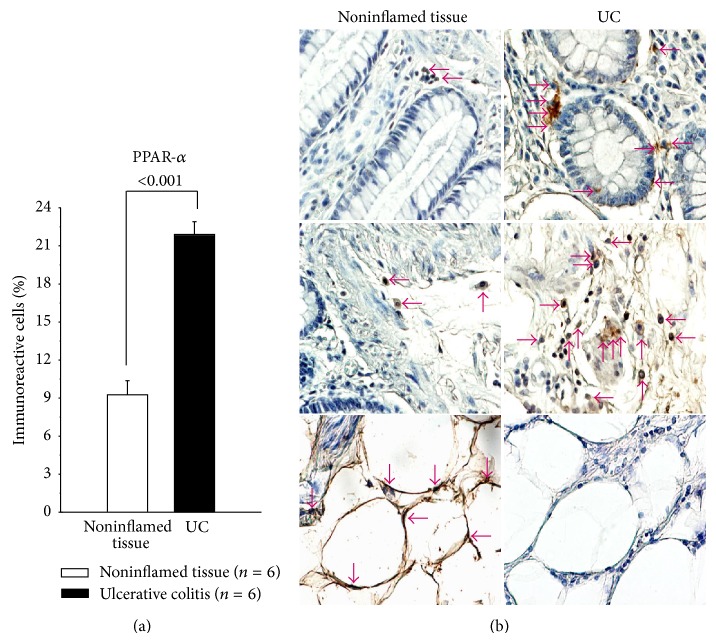
Percentage of PPAR*α*-expressing cells in active UC patients. Representative immunoperoxidase analysis in noninflamed colonic tissue and active ulcerative colitis tissue. Arrows depict immunoreactive cells in mucosa, submucosa, and adipose tissue. Original magnification was ×320.

PPAR*α*-producing cells were found mainly in mucosa, submucosa from patients with active UC versus controls (*P* < 0.001). The PPAR*α* in active UC was synthesized largely by epithelial cells, monocytes/macrophages, and lymphocytes, but not for adipose tissue (Figure 3).

In the same vein, PPAR*β* and PPAR*γ* protein expression in colonic tissue from active UC patients was plentiful compared with noninflamed colonic tissue.

**Figure 4:**
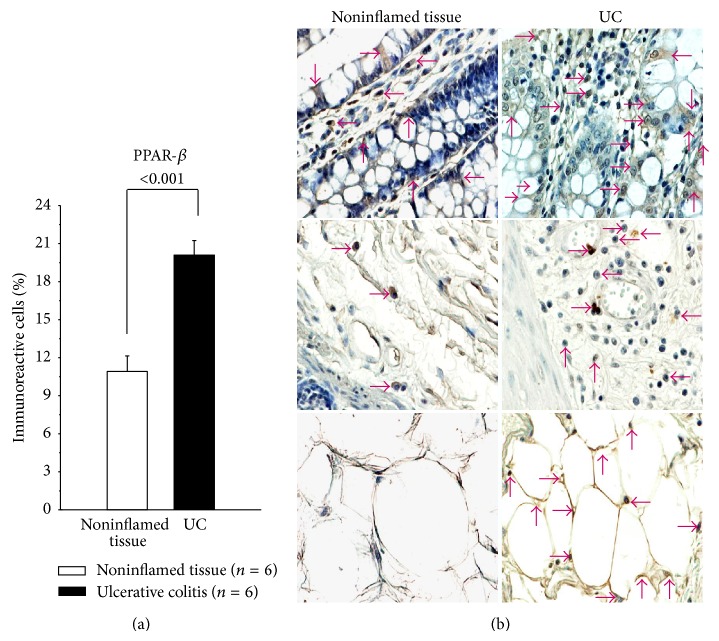
Percentage of PPAR*β*-expressing cells in active UC patients. Representative immunoperoxidase analysis in noninflamed colonic tissue and active ulcerative colitis tissue. Arrows depict immunoreactive cells in mucosa, submucosa, and adipose tissue. Original magnification was ×320.

PPAR*β*-producing cells were localized mainly in mucosa, submucosa, and perivascular inflammatory infiltrate. PPAR*β* in UC patients was produced by lymphocytes, monocytes/macrophages, fibroblasts, and endothelial cells (Figure 4). The percentage of immunoreactive cells was higher in patients with active UC compared to the noninflamed control group (*P* < 0.001).

**Figure 5:**
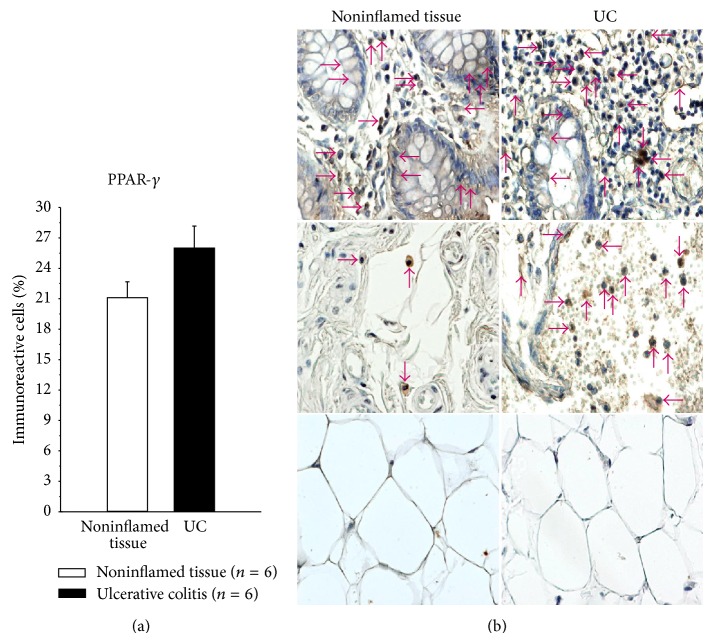
Percentage of PPAR*γ*-expressing cells in active UC patients. Representative immunoperoxidase analysis in noninflamed colonic tissue and active ulcerative colitis tissue. Arrows depict immunoreactive cells in mucosa, submucosa, and adipose tissue. Original magnification was ×320.

PPAR*γ* in patients with active UC was synthesized by epithelial cells, monocytes/macrophages, and lymphocytes, but not in adipose tissue and perivascular inflammatory infiltrate (Figure 5).

## 4. Discussion

Peroxisome proliferator-activated receptor gamma (PPAR*γ*) is member of a family of nuclear receptors that interacts with nuclear proteins acting as coactivators and corepressors. Colon is a major tissue which expresses PPAR*γ* in epithelial cells and, to a lesser degree, in macrophages and lymphocytes and plays a role in the regulation of intestinal inflammation [8]. PPAR*γ* ligands have beneficial effects in different models of experimental colitis, with possible implication in the therapy of IBD. Functional, biological, pharmacological, and chemical evidence has shown that aminosalicylates are a new functional synthetic ligand for PPAR*γ* in colonic epithelial cells [9]. PPAR*γ* is indeed the key receptor mediating the 5-ASA activity, by inhibiting several key target genes such as NF-*κ*B, signal transducers, and activators of transcription.

This is the first study, to our best knowledge, in patients with UC where PPAR*α* was studied. The PPAR*β* and PPAR*γ* were evaluated at gene and protein expression level in intestinal tissue regarding its clinical activity. Thus, we found a decrease of PPAR*α* and PPAR*γ* mRNA levels in active UC compared with noninflamed donors as previously described [10]. Conversely, percentage of PPAR*α* and PPAR*γ* immunoreactive cells in UC patients was increased compared with noninflamed tissues. This can be explained because the mRNA presence not always is related to protein expression (correlation between the RNA and protein profile is ~33 and 40%). It depends on half-life of different proteins (minutes to days) and/or posttranscriptional modification, RNA transport mechanisms, mRNA degradation (2–7 hrs), complex gene regulatory mechanisms, RNA processing, alternative splicing, RNA stability, and so forth [10]. Besides, samples used to determine mRNA expression are only from colonic mucosa, due to the fact that tissue was obtained by colonoscopy. Nonetheless for immunohistochemistry all colonic tissue was employed.

Previously, we showed decreased gene expression of PPAR*γ* in colonic biopsies from patients with active UC and its expression was negatively correlated with the severity of endoscopic disease activity [11]. Another study also has reported an impaired expression of PPRA*γ* in patients with UC [12].

According to our results, the use of rosiglitazone could induce higher expression of PPAR*γ* in the mucosa of the colon in order to decrease disease activity in patients with UC as it was previously demonstrated in a randomized placebo-controlled trial [13].

We observed an increase of PPAR*α* gene expression in patients with UC in remission and under treatment of monotherapy with 5-aminosalicylates compared to those who received any other combined therapy, suggesting a protective role of PPARs in patients with UC due to its anti-inflammatory activity. Nevertheless, no significant associations were found in relation to gene expression and other demographic and clinical characteristics.

We also found an increase of PPAR*γ* gene expression was associated with mild clinical course of the disease, characterized by initial activity and then long-term remission for more than 5 years. These findings suggest that patients with high gene expression of PPARs have a better response to medical treatment and mild clinical course of disease.

Even though this is a descriptive study, the findings are of interest; as far as we know, it is the first depiction of the association of PPARs gene expression with clinical therapy in UC. Additional studies about PPARs in the gut mucosal inflammatory process and the therapy with 5-aminosalicylates can begin to support the protective role of PPARs family in the intestinal inflammatory process [14, 15].

In conclusion, PPAR*α* and PPAR*γ* could be used as potential markers of better response to medical treatment based on 5-aminosalicylates and mild clinical course of UC.

## Figures and Tables

**Table 1:** Demographic data and disease characteristics from ulcerative colitis patients and controls.

	Control	Remission UC	Active UC
Mean age (years)	44.4	40.4	39.4
Gender			
Female	19	9	15
Male	12	11	11
Clinical course			
Initially active and then inactive		10	6
Mild intermittent activity: <2 relapse/year		10	19
Intense intermittent activity: >2 relapse/year		0	0
Continuous activity (refractory to treatment)		0	1
Extension			
Pancolitis (*n*)		9	16
Left-sided colitis (*n*)		5	4
Proctosigmoiditis (*n*)		6	6
Extraintestinal manifestation			
Present (*n*)		8	9
Absent (*n*)		12	15
Treatment			
Monotherapy with 5-aminosalicylates (*n*)		12	7
Combined therapy (*n*)		6	13
Response to treatment			
Present (*n*)		17	19
Absent (*n*)		3	7

## Abbreviations

IBD: Inflammatory bowel disease
UC: Ulcerative colitis
RT-PCR: Real-time polymerase chain reaction
PPARs: Peroxisome proliferator-activated receptors.
